# Are Aloes Admissible in Colics

**Published:** 1892-02

**Authors:** H. R. Macauley

**Affiliations:** Indianapolis


### ARE ALOES ADMISSIBLE IN COLICS.

BY H. R. MACAULEY, V. S.

I do not think I am wrong in saying, that at least seventy-five per cent. of the veterinary practitioners of to-day, when called in to see a case of colic, give, in conjunction with an anodyne, an aloes bolus, containing from five to eight drachms of aloes, and in this practice they are following out the mode of treatment prescribed by nearly all the authors of standard works on veterinary medicine. Professors Williams, Robertson, Gamgee, Greswell in England, and Professor Law in this country, all prescribe this in their works, but, with this array of literature in its favor, is it proper and scientific treatment. With such a drug as aloes there can be no middle course, it must be either beneficial or the reverse, and this being so, it behooves us, to seriously consider for ourselves whether or not in administering it we are doing rightly, and not follow blindly and unreasoningly, in the old rut of by-gone practice. For my part from personal observation and study I am greatly averse to its use in colic cases. Of course this dose of aloes is given for its cathartic effects, and these effects I take it, are intended to remove the irritant of whatever description it may be, that is causing the trouble. Now, I am a firm believer in the old adage, "remove the cause and the effects will cease," and if the aloes really fulfilled this purpose it would be an excellent remedy, but does it do this?

The better to understand the real action of aloes in colics, it might be advisable to divide our colics, whether spasmodic or flatulent into three classes, according to the length of time they last and see what the action of the aloes is in each of these classes.

Class I Those colics that are under control in from four to six hours or less.

Class II Those colics that last from eight to sixteen hours.

Class III Those colics going beyond sixteen hours.

In Class I we have cases that have promptly given way to treatment, the bowels have gradually regained their ordinary tonicity, the irritant, no matter what its nature, has lost its irritating effects and the horse is practically well and fit shortly to go to its accustomed work. Now what good does an aloes ball do in such a case? Ordinarily it takes from twenty to twenty-four or six hours for its action on the bowels to be first noticeable, so that we find the purgation commencing, about the time the horse should have been able to go to work, had the aloes not been given him. The purgation will last from twelve to twenty hours, so that virtually with our aloes we have created a new trouble, that considerably weakens the system and the horse has to remain in the stable, some two days or so, longer than was necessary, in order to recover from this new trouble. This being the case I leave it to the reader to say whether aloes has been beneficial or injurious in this class.

In Class II those colics lasting from eight to sixteen hours, we have to deal with a much more serious condition of affairs than in Class I. Here, the animal has undergone an amount of physical suffering, that has greatly weakened the whole system, and particularly must this weakness be apparent, at the seat of the trouble. Let us remember that the involuntary muscles of the intestines are governed pretty much by the same laws as govern the muscles that are subject to our will. The result of the continued irritation has been a congestion of the bloodvessels, an extra degree of sensitiveness, a pain, and the longer the irritant has been there the greater has been the strain on the weakened muscles and the greater the congestion. Now if at the sixteenth hour the pain has been conquered, we know that the irritating cause can no longer exist, and the great essential requisite for recovery of such a case is rest. Give the bowels rest, and nature will do her part in bringing the congested portion back to its normal condition. But what will the dose of aloes do? Their specific action being on the bowels we find the weakened, congested organs called upon to perform a great deal more peristaltic work than they have ordinarily to do when healthy, so that rest the greatest of all healers in such cases as class two, is denied, and extra stimulation produced. What good results can we expect from this treatment? The healthy bowel will respond readily to the action of the aloes and the ingesta is forced down to the weak and tender part, and the result is either one of two things. If the weakened bowel still retain sufficient strength to respond to this emergency, the ingesta is passed on, if on the other hand, this portion is too much weakened to respond to the stimulation, the ingesta forced here acts as an irritant, and we again have colicy pains, and we can consider ourselves indeed fortunate if we do not have a case of enteritis as a result and the question is, are we at liberty to use a drug whose action leaves even a possibility of such an untoward result? For a moment, let us look at the purgation that ensues should the bowel still respond to the stimulation. If the pain has been very intense, we find the whole system weak, and as a consequence, the purgation extends over a considerable length of time, sometimes it causes superpurgation, the nervous mechanism of the intestines in particular, and the system generally, being so much below par from the previous trouble that the inhibitory or regulating nerves of the intestines, from sheer exhaustion, are unable to regain control over the excretory glands, and at best, we have a very weakened animal if not a troublesome case of superpurgation. The patient in any case is laid up from work as in class one, but for a longer period of time. The more greatly weakened system taking longer to recuperate.

In Class III it might benefit us were we to look into the general medication the patient has been receiving. In the great majority of cases that have lasted this length of time, without relief being obtained, we have fallen back to our old standby opium, or its derivative, morphine, and their anodyne effects. Now we know that one of the chief actions of this powerful drug is its action on the peristalsis of the bowels. It checks peristalsis, and in this respect aloes is directly antagonistic, this being so, can one imagine any treatment more irrational or unscientific, here we have the congested and weakened intestines between two opposing fires. The opium while tending to sooth the whole system has its localized action on the intestines and giving them rest (which in this case means life), and aloes while nauseating the system calls upon the intestines for more work thus counteracting the benefits we might have expected from the anodyne.

In classes one and two the anodyne medication had ceased hours before the aloes commenced to act, and although the effects of this drug in these two cases were bad enongh they were not nearly so injurious as they are in cases coming under class three.

As a general thing we find cases that have lasted as long as class three to be in considerable pain about the time the aloes would act, although we sometimes find the pain not very severe, but continuous, the horse pawing incessantly. In this case we know the trouble does not extend over a very great extent of surface and most likely is imagination. The intensity of the pain being our guide as to the extent of congestion. How does the aloes ball act in these cases? My experience is that we will have no visible action whatever—why? If the trouble is due to invagination the feces accumulate there and can get no farther, merely intensifying the trouble. If there has been a very great amount of opium given it may be it has counteracted the effect of the aloes. If the case is one that we were afraid would end in enteritis we have virtually sealed its fate for reasons before explained. With these facts before us can we conscientiously advocate the use of aloes in colics? I have long ago given it up as incorrect, but my attention has been very forcibly directed to this lately, in a case, where I was called in for consultation. The attending surgeon had great faith in aloes and about three aloes pills were given, the result being that the patient died on the third day. The great object being with the surgeon to force a "passage" instead of giving rest.

I do not wish to leave the impression that I am negativing the use of all purges in colics. Some, such as pilocarpine and eserine I think particularly good in many cases, for as a rule, these act promptly, and by this prompt action, good is accomplished, while with aloes it is entirely different. In this last case it is a random shot as we are never certain how long a case of colic will last and not knowing this we cannot know whether the effects of the aloes will be injurious or harmless and unnecessary.

INDIANAPOLIS.

